# The Effect of a High-Fat Diet on the Fatty Acid Composition in the Hearts of Mice

**DOI:** 10.3390/nu12030824

**Published:** 2020-03-20

**Authors:** Alicja Pakiet, Agnieszka Jakubiak, Paulina Mierzejewska, Agata Zwara, Ivan Liakh, Tomasz Sledzinski, Adriana Mika

**Affiliations:** 1 Department of Environmental Analysis, Faculty of Chemistry, University of Gdansk, Wita Stwosza 63, 80-308 Gdansk, Poland; alicja.pakiet@phdstud.ug.edu.pl (A.P.); agata.zwara@phdstud.ug.edu.pl (A.Z.); 2 Tri-City Academic Laboratory Animal Centre - Research & Services Centre, Medical University of Gdansk, 80-210 Gdansk, Poland; a.jakubiak@gumed.edu.pl; 3 Department of Biochemistry, Faculty of Medicine, Medical University of Gdansk, Debinki 1, 80-211 Gdansk, Poland; paulina.mierzejewska@gumed.edu.pl; 4 Department of Pharmaceutical Biochemistry, Faculty of Pharmacy, Medical University of Gdansk, Debinki 1, 80-211 Gdansk, Poland; liakh_ivan@mail.ru (I.L.); tsledz@gumed.edu.pl (T.S.)

**Keywords:** heart, high-fat diet, fatty acids, cell membrane, sphingolipids, phospholipids, polyunsaturated fatty acids

## Abstract

The Western diet can lead to alterations in cardiac function and increase cardiovascular risk, which can be reproduced in animal models by implementing a high-fat diet (HFD). However, the mechanism of these alterations is not fully understood and may be dependent on alterations in heart lipid composition. The aim of this study was to evaluate the effect of an HFD on the fatty acid (FA) composition of total lipids, as well as of various lipid fractions in the heart, and on heart function. C57BL/6 mice were fed an HFD or standard laboratory diet. The FA composition of chow, serum, heart and skeletal muscle tissues was measured by gas chromatography–mass spectrometry. Cardiac function was evaluated by ultrasonography. Our results showed an unexpected increase in polyunsaturated FAs (PUFAs) and a significant decrease in monounsaturated FAs (MUFAs) in the heart tissue of mice fed the HFD. For comparison, no such effects were observed in skeletal muscle or serum samples. Furthermore, we found that the largest increase in PUFAs was in the sphingolipid fraction, whereas the largest decrease in MUFAs was in the phospholipid and sphingomyelin fractions. The hearts of mice fed an HFD had an increased content of triacylglycerols. Moreover, the HFD treatment altered aortic flow pattern. We did not find significant changes in heart mass or oxidative stress markers between mice fed the HFD and standard diet. The above results suggest that alterations in FA composition in the heart may contribute to deterioration of heart function. A possible mechanism of this phenomenon is the alteration of sphingolipids and phospholipids in the fatty acid profile, which may change the physical properties of these lipids. Since phospho- and sphingolipids are the major components of cell membranes, alterations in their structures in heart cells can result in changes in cell membrane properties.

## 1. Introduction

The Western diet (WD) is characterized by overeating and an especially high intake of simple carbohydrates and saturated fats. Combined with a lack of physical activity, the WD can lead to obesity and related comorbidities, especially cardiovascular diseases (CVD) [1,2]. Recent data indicate that there is practically no organ or system that would not be affected by the WD [3]. The first target of the WD is the digestive system [4,5,6]. The WD leads to microbiome changes [7,8], which may be a factor in the development of sporadic colon cancer [9,10] or inflammatory bowel disease [11]. The WD also affects the liver [12], which promotes the development of non-alcoholic fatty liver disease and [13] liver fibrosis [14] and disrupts bile acid synthesis [15]. Acting on the brain, the WD causes cognitive impairment [16,17,18], increases neuroinflammation and memory deficits [19], raises the risk of dementia [20], Alzheimer’s disease [21], and autism spectrum disorders [22], and causes changes in the retina [23]. Changes in immune status, as a result of the WD, lead to sepsis [24], auto-inflammation [25], hypersensitivity [26], breast [27] and skin [28] inflammation, acne [29], and a decrease in infection control [30]. A WD leads to decreased fertility [31], foetal changes during pregnancy [32] and after childbirth [33,34], affects sex hormones [35] and causes negative effects on the kidneys [36,37].

However, in terms of mortality, the greatest danger to human health is the fact that the WD affects the cardiovascular system [2,38,39,40]. The World Health Organization claims that CVD is the leading cause of death in the world, with an estimated 17.9 million deaths per year (31% of all deaths in the world), which creates a huge burden on the health care system and the economy. The American Heart Association reports that 74% of the risk of stroke can be attributed to behavioural risk factors, including unhealthy diets [41]. A high-fat diet (HFD) is a well-established rodent model of the WD. Existing theories describing the relationship between HFDs and cardiovascular disorders mainly describe mechanisms for the development of structural and metabolic changes and the response to increased levels of inflammation during HFD in heart tissue [36,42,43,44,45,46]. An important role is attributed to intensified oxidative stress during HFD intake and subsequent activation of the inflammasome complex, which leads to activation and secretion of cytokines (IL-1β and IL-18), inducing sterile inflammation [47,48]. While the majority of studies suggested deterioration of cardiac contractility consequent to HFD [49,50,51,52,53], Mourmoura et al. [54] found an improvement of cardiac function after 3 months of feeding rats with HFD containing a high proportion of SFA. This underpins the importance of further studies to clarify this issue.

One theory explaining the adverse effects of a HFD on the heart is that decreased expression of genes of antioxidant enzymes, such as malic enzyme, and activation of caspase-3 in the heart leads to apoptosis due to oxidative stress and disruption of the anaplerotic flow of the Krebs cycle, respectively [55]. Additionally, enhanced β-oxidation of fatty acids (FAs) with HFD intake leads to the formation of reactive oxygen species and lipid peroxidation, which contributes to a change in mitochondrial function [56]. According to another theory, a HFD initiates cascading reactions in which increased carnitine palmitoyl transferase (CPT1) and uncoupling protein (UCP2) contents lead to reduced plasma membrane glucose transporter (GLUT4) and peroxisome proliferator-activated receptor gamma coactivator 1-alpha (PGC-1α) contents, which ultimately leads to reduced mitochondrial biogenesis and uncoupling of heart bioenergetic metabolism [57,58]. Overload, hypertrophy and a decrease in left ventricular conduction may be associated with exposure to free fatty acids (FFAs) and the accumulation of triglycerides in and around the myocardium, as well as a significant generalized excess of ectopic fat [59].

The harmful effects of a HFD on the heart are also associated with changes in cardiac mitochondrial membrane fluidity after a decrease in myocardial palmitoleoyl-CoA (16:1-CoA) content, wherein an enhanced activity of FA elongase and desaturase leads to increased hepatic lipogenic capacity [50]. Some authors described reduced 16:1-CoA after long-term HFD intake. Recent data also indicate that the FA composition of membrane phospholipids has a great influence on the properties of membranes and that the degree of unsaturation of membrane phospholipids correlates with contractile dysfunction [51]. Both exogenous saturated FAs (SFAs) and monounsaturated FAs (MUFAs) from the HFD cause different degrees of adverse myocardial changes as a consequence of the change in the levels of SFAs and MUFAs in the membrane phospholipids [51]. However, the authors of the above-mentioned paper did not study changes in polyunsaturated FA (PUFA) levels, although it is known that a high food intake of PUFAs reduces the risk of CVD [38,60]. Thus, the aim of this study was to evaluate the effect of an HFD on the whole FA composition, including PUFAs, in mouse hearts, both in all lipids and in specific lipid fractions.

## 2. Materials and Methods

### 2.1. Animals and Treatment

The animal study protocol was approved by the Local Ethics Committee for Experiments on Animals in Bydgoszcz (approval number 47/2016). Individually marked 6-week-old male mice (strain C57BL/6) from Tri-City Animal Laboratory Research and Service Center, Medical University of Gdansk, with an average body weight of 21.8 ± 1.1 g, were randomly assigned to two experimental groups (two cohorts—one for tissue collection and second for cardiac function assessment; 10 controls and 10 mice treated by HFD in each cohort). In each cohort, ten animals were fed a standard diet (SD) (Altromin C 1090 – 10) with energy from 11% fat, 24% protein and 65% carbohydrates (ME 14.6 MJ/kg), while the other 10 mice were provided a high-fat diet (HFD) (Altromin C 1090 – 60) containing 60% energy from fat, 16% protein, and 24% carbohydrates (ME 21.1 MJ/kg). The animals were housed in polysulfone cages in a room controlled for temperature (22 ± 2°C), humidity (55 ± 10%), and light cycle (12 h light/12 h dark). The air was exchanged ≥12 times per hour. The mice had access to chow and water *ad libitum*. Body weight and chow intake were measured weekly. After 19 weeks of experiments, the animals were sacrificed, and blood and organs/tissues were harvested. Hearts and sections of approximately 500 mg of skeletal muscle were immediately frozen in liquid nitrogen. Blood was centrifuged at 3000× *g* for 15 min at 4 °C, and serum was aliquoted. All samples were stored at –80 °C until analysis. Triacylglycerols (TAGs) and total protein levels were estimated using a laboratory analyser (XL-100, Erba Diagnostics Mannheim GmbH, Mannheim, Germany).

Plasma non-esterified fatty acid was measured using free fatty acid (FFA) Kit (MAK044, Sigma-Aldrich, St. Louis, MO, USA) according to the manufacturer’s protocol. Briefly, 50 µL of a serum sample was added to individual plate wells. A quantity of 2 μL of Acyl-CoA synthase was added to each well and incubated at 37 °C for 30 min in the dark. Then, 50 μL of the master reaction mixture consisting of 44 μL of fatty acid assay buffer, 2 μL of fatty acid probe, 2 μL of enzyme mix and 2 μL of enhancer were added and incubated under the same conditions. After that, absorbances for individual wells were read at 570 nm using a Synergy HT multi-detection microplate reader (BioTek, Winooski, VT, USA).

### 2.2. Cardiac Function Assessment

At the age of 24 weeks, mice were anaesthetized with ketamine (100 mg/kg) and xylazine (10 mg/kg) intra-peritoneally. After chest hair removal, animals were placed on a heated platform to maintain the body temperature at 37 °C. Transthoracic echocardiography was performed with a Vevo 1100 (VisualSonics Inc, Toronto, Canada) equipped with a 40-MHz linear array transducer. Images were acquired at a frame rate consistently above 200 frames/s. The transducer was placed above the anterior chest wall and directed towards the ascending aorta in 2D mode, which was next switched to Doppler flow velocity mode. The readings were recorded and used directly or applied for aortic valve area (AVA) calculation. The AVA was determined with the continuity equation. Haemodynamic parameters, including stroke volume (SV), left ventricular (LV) ejection fraction (EF) and cardiac output (CO), were collected.

### 2.3. Lipid Analysis

#### 2.3.1. Total Lipid Extraction

Lipids were extracted from tissue samples with a chloroform–methanol mixture (2:1, v/v) according to the method published by Folch et al. [61]. The chloroform phase was collected, divided into two parts, dried under a nitrogen stream and stored at −20°C for further analysis.

#### 2.3.2. Solid Phase Extraction

Aliquots of total lipid extracts were fractionated on aminopropyl solid phase extraction (SPE) columns (Strata^®^ NH2 500 mg, Phenomenex^®^, Torrance, CA, USA) in accordance with two procedures. Method I, by Kaluzny et al. [62], allowed for the collection of FFAs, phospholipids (PLs) and acylglycerols (AGs). Two milligrams of dried extracts were reconstituted in chloroform and loaded onto SPE columns preconditioned with 2 × 2 mL of n-hexane. The phases were then eluted with 6 mL chloroform-isopropanol (2:1, v/v) to obtain neutral lipids (NLs), 6 mL 2% acetic acid in diethyl ether (v/v) to obtain FFAs, and 6 mL methanol to obtain PLs; all fractions were dried under a nitrogen stream. The NL fraction was dissolved in n-hexane and loaded onto a new SPE cartridge and fractionated with 6 mL n-hexane to obtain cholesteryl esters, which were discarded, 9 mL methylene chloride:diethyl ether-n-hexane (10:1:89, v/v/v) to obtain TAGs, 18 mL 5% ethyl acetate in n-hexane (v/v) to obtain cholesterol, which was discarded, 6 mL 15% ethyl acetate in n-hexane (v/v) to obtain diacylglycerols (DAGs) and 6 mL chloroform–methanol (2:1, v/v) to obtain monoacylglycerols (MAGs). The MAG, DAG and TAG phases were combined into an AG mixture, and all fractions were dried under a nitrogen stream.

Method II, by Bodennec et al. [63], used 1.5 mg lipid extract reconstituted in chloroform and loaded onto aminopropyl columns preconditioned with 5 mL of n-hexane. Samples were eluted with 5 mL 15% ethyl acetate in n-hexane (v/v) to obtain NLs without ceramides (Cer), MAGs and FFAs, 4 mL chloroform-methanol (23:1, v/v) to obtain Cer, 3 mL 5% acetic acid in diisopropyl ether (v/v) to obtain FFAs and α-hydroxy-FFAs, which were discarded, 11 mL acetone–methanol (9:1.35, v/v) to obtain glycosphingolipids (GSPLs), and 4 mL chloroform–methanol (2:1, v/v) to obtain sphingomyelins (SMs). All obtained fractions were evaporated to dryness under a nitrogen stream.

#### 2.3.3. Fatty Acid Hydrolysis and Derivatization

All fractions collected after SPE and total lipid extracts were prepared for GC–MS analysis as follows. One millilitre 0.5 M KOH in methanol was added, and samples were hydrolysed at 90 °C for 3 h. Then, mixtures were acidified with 0.5 mL 5 M HCl, 1 mL of water was added, FAs were extracted with 3 × 1 mL of n-hexane, and the organic phase was evaporated to dryness. Derivatization to FA methyl esters (FAMEs) was achieved with a 10% boron trifluoride–methanol solution at 55 °C for 1.5 h. After incubation, 1 mL water was added, and FAMEs were extracted with 1 mL n-hexane thrice and dried under a nitrogen stream.

#### 2.3.4. GC–MS Analysis

The FAME composition of the obtained samples was determined by using a QP-2010SE GC-EI-MS (Shimadzu, Kyoto, Japan). The chromatographic separation was conducted on a Zebron ZB-5MSi capillary column (30 m length × 0.25 mm i.d. × 0.25 μm film thickness, Phenomenex^®^, Torrance, CA, USA) with helium as the carrier gas (head pressure of 100 kPa). The GC oven temperature was programmed from 60 to 300°C (4°C/min) with an overall run time of 60 min. The electron impact source for mass spectrometric detection was operated at 70 eV. Acquisition of mass spectra was conducted in full scan mode with a mass scan range of m/z 45–700. 19-Methylarachidic acid was used as an internal standard. Peak identification was aided by the use of reference standards (37 FAME Mix, Sigma-Aldrich, St. Louis, MO, USA) and the NIST 2011 reference library.

### 2.4. Lipid Peroxidation

Lipid peroxidation was measured using the TBARS Assay Kit (cat. no. 10009055, Cayman Chemical Company, Ann Arbor, MI, USA). This method is based on the colorimetric determination of coloured adducts formed by the reaction of malondialdehyde (MDA) and thiobarbituric acid (TBA) at high temperatures (90–100 °C) under acidic conditions. Briefly, approximately 25 mg of heart tissue was added to 250 μL RIPA buffer (containing selected protease inhibitors), the tissues were homogenized on ice, and the tube was centrifuged at 1600× *g* for 10 min at 4 °C. One hundred microlitres of the supernatant were removed for analysis and mixed in a 5 mL vial with 100 μL SDS solution, after which 4 mL colour reagent was added, and the vials were placed in boiling water for 1 h. After an hour, the vials were placed in an ice bath and incubated for 10 min to stop the reaction. After centrifugation of the vials for 10 min at 1600× *g* and 4 °C, 150 μL aliquots of the samples (in duplicate) were loaded from each vial into the plate, and the absorbance was read at 532 nm. Determination of concentration was carried out using a calibration curve, which was obtained using the standard provided in the kit.

### 2.5. Free Cholesterol Measurement

Free cholesterol content in mouse heart was measured using reagent for the quantitative in vitro determination of free, unesterified cholesterol in serum or plasma (Greiner Laboratories GmbH, Germany) according to the manufacturer’s instructions. The colorimetric indicator is quinoneimine dye, which is formed from the catalytic action of peroxidase from 4-aminoantipyrine, phenol and hydrogen peroxide (Trinder reaction). Briefly, 10 μL of the supernatant is added to 1 mL of reagent, after which the samples are incubated for 20 min at 37 °C. After that, absorbance was determined spectrophotometrically at 546 nm using a Synergy HT multi-plate microplate reader (BioTek, Winooski, VT, USA).

### 2.6. Protein Carbonyl Groups Determination

Protein carbonyl content in mouse heart was measured using The Protein Carbonyl Content Assay Kit (Sigma-Aldrich, St. Louis, MO, USA, MAK094) according to the manufacturer’s instructions. The method is based on the derivatization of protein carbonyl groups with 2,4-dinitrophenylhydrazine (DNPH), which leads to the formation of stable dinitrophenyl adducts (DNP) of hydrazone, which is further detected spectrophotometrically at 375 nm. Briefly, 100 μL of a DNPH solution was added to 100 μL of the homogenate and incubated for 10 min at room temperature. Then, 30 μL of a 100% TCA solution was added to each sample, shaken and incubated on ice for 5 min. After centrifugation, the pellet was washed twice with 500 μL of acetone. The resulting precipitate was dissolved in 200 μL of a 6 M guanidine solution and the absorbance was measured at 375 nm using a Synergy HT multi-plate microplate reader (BioTek, Winooski, VT, USA). In addition, the amount of total protein was determined using a laboratory analyser (XL-100, Erba Diagnostics Mannheim GmbH, Mannheim, Germany), and the results are presented as nmol/mg of total protein.

### 2.7. Statistical Analysis

The data are presented as the mean ± SD. Every sample was run in duplicate. For normally distributed data, the significance of differences between means was estimated with parametric Student’s t-tests. The data that did not follow a normal distribution underwent a non-parametric Mann–Whitney U test. The statistical analysis was performed with Sigma-Plot 11 software (Systat Software, Inc. 2008, San Jose, CA, USA).

## 3. Results

### 3.1. Dietary-Induced Obesity

The 19-week-long treatment of mice resulted in a gradual, almost 30% increase (*p* < 0.001) in body weight of HFD-fed mice (final weight: 42 ± 5.3 g) compared to that of the SD-fed mice (final weight 30 ± 1.6 g) [64]. However, the mean weight of the animals’ hearts did not differ significantly between the SD and HFD groups (218 ± 14 g and 214 ± 26 g, respectively). Likewise, the total protein concentration in the hearts was similar for HFD-fed mice (164 ± 31 g/g of wet tissue) and SD-fed mice (157 ± 12 g/g of wet tissue). To assess fat accumulation in the hearts, we measured the TAG content in tissue homogenates. The HFD-fed mice had 1.7 times more TAGs in the heart tissue (5.0 ± 1.7 mg/g of wet tissue) than that in the heart tissue of SD-fed mice (2.9 ± 0.5 mg/g of wet tissue) at *p* < 0.01. Moreover, mice fed with the HFD had significantly higher serum FFA concentrations than that of mice fed the SD (0.68 ± 0.34 vs 0.25 ± 0.12 mmol/L respectively, *p* < 0.001). We also measured free, non-esterified cholesterol in heart tissues and found no significant differences between HFD mice (3.62 ± 0.34 mg/g of wet tissue) and SD-fed mice (3.79 ± 0.51 mg/g of wet tissue, *p* = 0.381).

### 3.2. Cardiac Function Assessment

To characterise the implications of the HFD for cardiac function, we conducted two-dimensional echocardiographic measurements and examined the aortic valve flow velocity using Doppler ultrasound. These results are presented in Table 1. The in vivo analysis of the stroke volume and cardiac output did not differ between mice fed the two diets. However, the HFD caused a significant decrease in aortic valve area compared to that determined in the hearts of mice fed the SD. Furthermore, the aortic valve flow velocity (Vmax) tended to be higher in HFD group (*p* = 0.056), and a trend was observed for a decrease in the cell ventricular ejection fraction in comparison to that of the control (Table 1)

### 3.3. Changes in Total FA Profiles

A high-fat diet is one of the experimental models of the Western diet, and Table 2 shows that high-fat chow contains much higher levels of SFA than control chow, which is a characteristic feature of the Western diet. The HFD diet resulted in a significant elevation of the total FA concentration in the serum of HFD-fed mice compared to that in the serum of SD-fed mice (4.35 ± 0.77 and 2.65 ± 0.49 µg/mL, respectively; *p* < 0.05). The concentrations of all major FA groups in the sera of animals, namely, SFAs, MUFAs and PUFAs, and both *n*-3 and *n*-6, were increased in the HFD group, which corresponded to the higher overall concentrations of these groups in the HFD chow (Table 2). Among PUFAs, eicosapentaenoic acid (EPA, 20:5 *n*-3) was an exception, as its content in HFD serum was two-fold lower (*p* < 0.001), despite a high concentration of this acid in the HFD chow (Table 2). We also observed an expected increase in oleic acid (18:1), which was the most abundant MUFA, and surprisingly, an almost two-fold decrease in the serum palmitoleic acid (16:1) concentration in HFD-fed mice, even though this FA chow content was over eight times higher in the HFD chow (Table 2).

**Figure 1:**
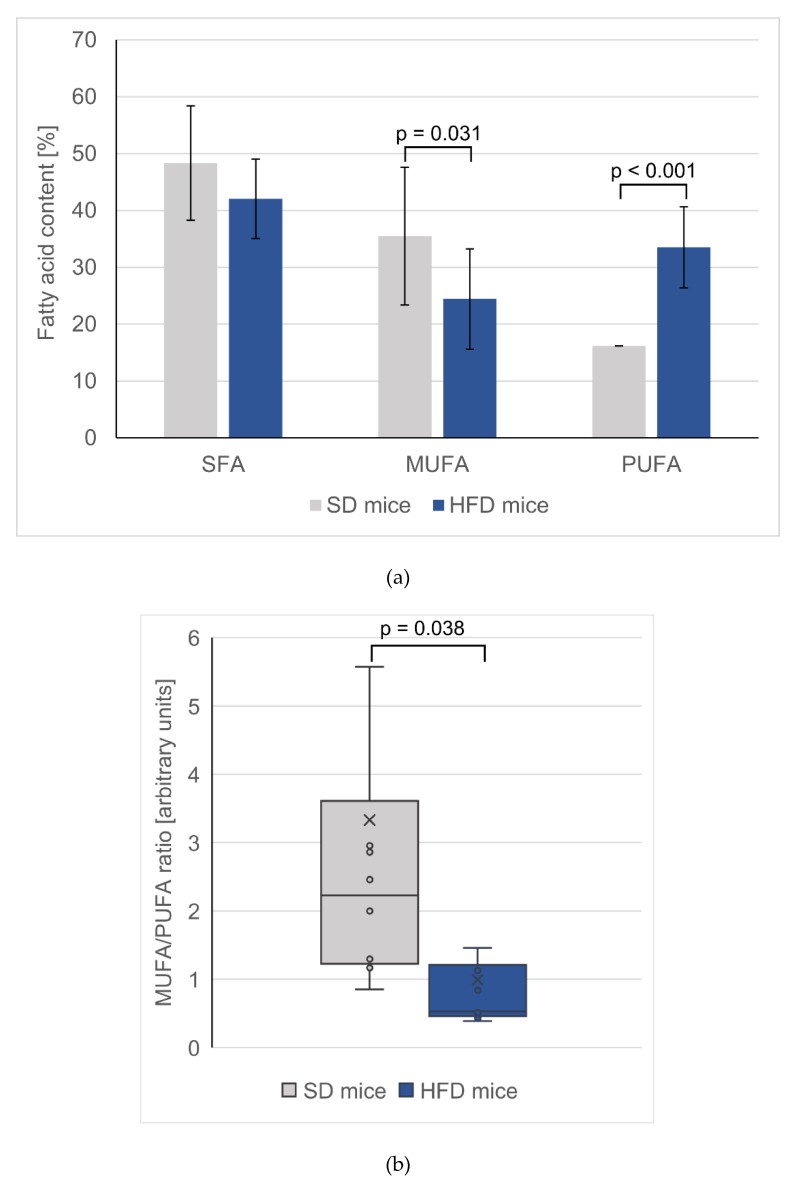
(**a**) Total saturated, monounsaturated and polyunsaturated fatty acids content in hearts. Values are mean ± SD. (**b**) Box-plot of monounsaturated to polyunsaturated fatty acid ratio in mice hearts. SD – mice fed standard diet; HFD – mice fed high fat diet.

In both the hearts and skeletal tissues of HFD-fed mice, the changes in PUFAs followed similar trends as those observed in the serum; that is, the levels of total *n*-3 and *n*-6 PUFAs increased (Table 2). Interestingly, the changes in *n*-3 PUFA levels were much more pronounced in the heart than in the skeletal muscles. The *n*-3 PUFA content in the hearts of HFD-fed mice was 2.6 times greater than in the hearts of SD-fed mice (*p* < 0.001), whereas in the skeletal muscle, a nonsignificant difference of only 50% was observed. The increased content of particular FAs was significant in the hearts for almost all measured PUFAs, with the exception of EPA and eicosatetraenoic acid (ETA, 20:4 *n*-3), whereas in the skeletal muscles, only some PUFAs changed significantly. In both tissues, the n-6 PUFA levels were almost two-times higher for mice fed an HFD than for mice fed the SD (*p* < 0.001), and there was a slight decrease in the SFA content. Notably, only in the hearts was the MUFA content in HFD-fed mice significantly lowered (approximately 30%), which was in contrast to the trend observed for the MUFA content in serum (Table 2). Additionally, both palmitoleic acid and oleic acid in HFD hearts were significantly lowered. The changes of total SFA, total MUFA, total PUFA are presented in Figure 1a and PUFA/MUFA ratio in the hearts of control and HFD mice are presented in Figure 1b. 

### 3.4. Fatty Acid Alterations in Specific Lipid Fractions of the Hearts

The use of SPE allowed for us to obtain several fractions containing both polar and nonpolar lipid groups from the hearts of animals, which were then used for the analysis of the various FA contents among each group. The first method of fractionation followed the protocol established by Kaluzny et al. [62], in which we separated lipids into FFAs, PLs and AGs. The second method of lipid separation by Bodennec et al. [63] yielded four fractions: NLs, Cer, GSPLs and SMs. 

**Figure 2:**
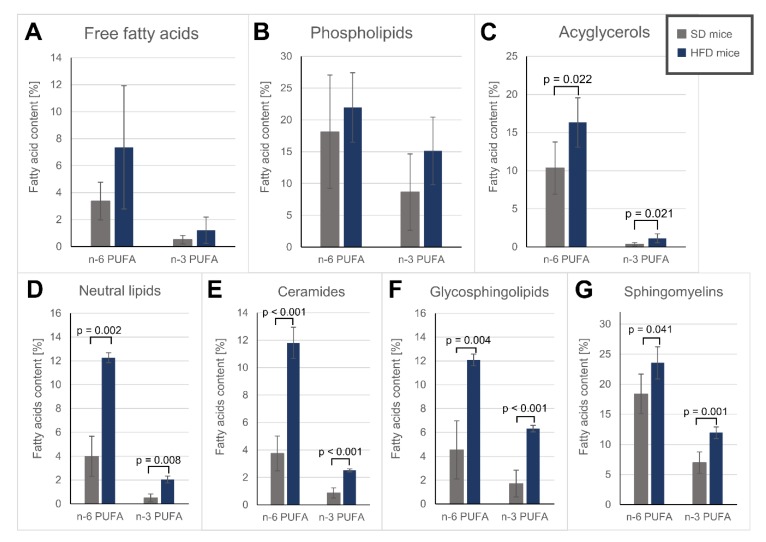
*n*-6 and *n*-3 polyunsaturated fatty acids content in different lipids fractions: (**A**) – free fatty acids; (**B**) – phospholipids; (**C**) – acylglycerols; (**D**) – neutral lipids; (**E**) – ceramides; (**F**) – glycosphingolipids; (**G**) - sphingomyelins; SD – mice fed standard diet; HFD – mice fed high fat diet.

Phospholipids (PL fraction), which are the main component of the lipid bilayer [65], were characterized by a slight, albeit non-significant, increase in PUFAs after HFD treatment (Table 3, Figure 2A–C). Among the obtained sphingolipid fractions, namely, Cer, GSPLs and SMs, the upregulation of the n-3 PUFA level was the largest in the GSPL fraction (3.7 times higher in HFD-fed mice than in SD-fed mice), whereas the *n*-6 PUFA level increased the most in the Cer fraction (3.1 times higher in HFD-fed mice than in SD-fed mice) (Figure 2D-G). EPA was significantly downregulated in most polar lipid fractions ( Table 3; Table 4) of the HFD-fed mice. Interestingly, arachidonic acid (ARA, 20:4 *n*-6) was the most abundant n-6 PUFA across all polar lipids except in the Cer fraction, where linoleic acid (LA, 18:2 *n*-6) predominated (Table 4), despite LA being supplied at higher concentrations in the diet. Both the Cer and GSPL fractions exhibited lower SFA contents in HFD-fed mice than in SD-fed mice, mostly due to the lowered stearic acid (18:0) content in the Cer fraction and the 16:0 decrease in the GSPL fraction (Table 4). On the other hand, SMs, an important component of lipid rafts [66], were characterized by a similar SFA content in both groups and a significant decrease in MUFA levels in the HFD-fed mice compared to those in the SD-fed mice (Table 4).

In both obtained non-polar groups, i.e., NLs and AGs, oleic acid was the most abundant FA, as MUFAs are major building blocks for AG synthesis [67], and the total MUFA percentage decreased in the hearts of HFD-fed mice (Table 3 and Table 4). Similar to the polar lipids we gathered, PUFA accumulation in the hearts of HFD-fed mice was apparent in the AG and total NL fractions (Table 3 and Table 4, Figure 2). Additionally, while we did not observe an increase in total SFAs, in the AG fraction, the stearic acid level increased almost two-fold in the HFD hearts.

### 3.5. Fatty Acid Oxidation and Protein Carbonylation Content

Lipid peroxidation in heart tissue, assessed on the basis of MDA formation, did not differ between the SD- and HFD-fed mice (8.41 ± 1.95 and 8.67 ± 1.29 µM MDA per g of total protein, respectively). Similarly, we did not find any difference in protein carbonyl groups level (1.59 ± 0.24 vs 1.52 ± 0.28 nmol/mg of total protein).

## 4. Discussion

Obesity is well known to be a risk factor for the development of cardiovascular diseases, in particular, coronary heart disease and heart failure [68,69]. Diet-induced obesity may lead to profound changes in heart lipid composition due to its limited capacity for de novo FA synthesis and, therefore, reliance on the exogenous supply of FAs [69]. Our study aimed to assess the influence of an HFD, a model of the Western diet, on heart function with regard to FA composition in different lipid groups. Opinion on the extent of the detrimental effect of HFD in the heart differs. Several studies imply that an HFD diet alone is not sufficient for inducing heart failure [44,70,71], while others report lipotoxic cardiomyopathy under long dietary regimens [49,50] or cardiac hypertrophy under specific FA contents of a HFD [72]. HFD treatment is also one of the established models for the induction of aortic valve disease [73,74,75]. HFD treatment altered aortic flow pattern [73]. Amendment of resting cardiac function did not identify significant deterioration consistent with earlier report [54]. Moreover, Mourmoura et al. [54] suggest an augmented inotropism after the 3-month HFD diet intake and increased cardiac output, resulting from an increased cardiac mechanical function. However, a trend for lower values in the HFD group may suggest that, in our study, under conditions of increased workload or with strain imaging, such dysfunction could manifest. We strived to explain the association of lipid alterations with observed changes in heart function considering 1) ectopic lipid accumulation, 2) oxidative stress and 3) FA content changes in different lipid groups.

Postnatal mammalian hearts depend on FA β-oxidation coupled with oxidative phosphorylation to generate ATP [76]. The heart uptakes FAs both in non-esterified form, as FFAs and as esterified, lipoprotein-bound species [77]. An increased consumption of fat causes the storage capacity of adipocytes to be exceeded, leading to the release of FFAs from adipose tissue and their elevated levels in serum [78], which was also confirmed in our study. The overabundance of circulating lipids may cause ectopic fat accumulation in the liver, skeletal muscle or heart [50,79], despite the heart’s preference for lipids as the energy substrate rather than for storage [80]. In HFD-fed mice, we observed that excess dietary supplementation of fat caused a higher cardiac content of TAGs, which is representative of neutral lipid accumulation. This result is consistent with that of previous studies [50,78] and is indicative of possible lipotoxicity [80]. Although TAGs themselves might not be toxic, they can indicate accumulation of other lipotoxic species, such as DAGs, which were reported to be accumulated under HFD intake [77,81]. Elevated circulation of FFAs was also a feature found in our study and was described by other authors in mice fed an HFD [78,82]. FFAs are utilized by cells for energy production via oxidative pathways or as substrates for complex lipid synthesis [83]. Du et al. [84] reported that increased FFA levels contribute to the overproduction of superoxide, which activates proinflammatory signals in aortic endothelial cells. One of the obesity hallmarks is the increase in oxidative stress and inflammation [68]. However, we did not observe differences in lipid peroxidation, nor in protein carbonylation between the HFD and SD groups, which may be attributed to the composition of the mouse chow used [72] or the duration of our study [50]. Similarly, the protein carbonyl content in heart tissue did not differ between SD and HFD mice. Moreover, Leger et al. [85] found that HFD led to decrease of cardiac oxidative stress and apoptosis rate in rats. This was associated with increased arachidonic acid proportion in membrane phospholipids. We also observed a trend to increase arachidonic acid in heart phospholipids but it did not reach statistical significance (Table 3).

Although there are some studies investigating the effects of dietary fat on rodent cardiac functions, most of them do not report specific FA profiles or do so only for total lipid FA profiles in heart tissue [50,53,72]. Alternatively, only FA profiles for one selected group of lipid species are presented [86,87,88]. Some studies focused mostly on SFAs and MUFAs [51], while PUFAs have not been widely reported. In contrast, in studies involving humans, the effects of PUFA supplementation on heart function were primarily considered [44].

In general, we found upregulation of both n-3 and n-6 PUFA series contents, as well as increased contents of most individual PUFAs in the heart muscle of mice fed an HFD, with the exception of significantly downregulated EPA (Table 2). This accumulation of specific PUFAs is consistent with previous studies; for example, mitochondrial phospholipids in hearts were shown to readily incorporate dietary DHA [89] or ARA [88]. One possible explanation for the elevated levels of long-chain PUFAs (ARA, DHA, etc.) is that these PUFAs can also be synthesized from exogenous 18-carbon PUFAs [90]. Another point worth considering is the different rates of FA oxidation (FAO). The readiness of cells for utilization of FA by β-oxidation, both mitochondrial and peroxisomal, seems to be dependent on both the degree of FA unsaturation and FA acyl chain length [91,92,93]. Experiments evaluating total body FAO [91,92] as well as FAO rates for different tissues, including the heart [93], point to a preference for unsaturated vs saturated FAs (oleic acid > stearic acid FAO rate). This may explain the significantly reduced MUFAs, which are preferred for β-oxidation over other FAs, in the hearts of HFD-fed mice. Among PUFAs, higher oxidation rates were observed for 18-carbon-chained PUFAs, especially for α-linolenic acid (ALA, 18:3 *n*-3), which is preferred for β-oxidation among other PUFAs [91] and may explain why we did not observe any ALA accumulation in hearts due to HFD treatment, in contrast to the observed accumulation of most other PUFAs (Table 2, Table 3 and Table 4). Another PUFA that did not accumulate in the hearts was EPA (Table 2, Table 3 and Table 4). However, it should be pointed out that the DPA content in the hearts of HFD-fed mice in our study increased three-fold (Table 2), and this FA is a metabolic intermediate between EPA and DHA, which may explain the depletion of EPA [90]. PUFAs can influence tissue function via their downstream metabolites. Moreover, *n*-3 PUFAs can exert positive effects on cardiac functions [94], which are mainly associated with anti-inflammatory metabolites of their enzymatic oxidation [95]. Increased arachidonic acid content in HFD-fed mouse serum and tissues may be explained both by the dietary supply and conversion of linoleic acid (LA, 18:2 *n*-6) [96] or, in the case of heart phospholipids, the preference of cardiac 1-acyl-sn-glycerol 3-phosphate acyltransferase (AGPAT) for ARA during phosphatidic acid synthesis [97]. While many ARA-derived eicosanoids have detrimental effects on cardiac health, some seem to be cardioprotective [98]. Keeping in mind that many LA-derived bioactive metabolites are, in fact, anti-inflammatory [96] and that an almost two-fold increase in the docosahexaenoic acid (DHA, 22:6 *n*-3) content was observed in HFD-fed mouse serum and heart, which is a precursor of anti-inflammatory docosanoids, the deleterious effects of EPA depletion may possibly be ameliorated by the increased content of these other PUFAs. Considering the association between inflammation and oxidative stress, the lack of oxidative stress in the hearts of HFD-fed mice found in our study may be the result of the accumulation of other anti-inflammatory *n*-3 PUFAs.

Since the changes in FA composition in various lipids can differentially impact cardiac function, we focused on trying to elucidate which lipid groups were most affected by an HFD. The SPE procedures allowed for us to obtain several lipid fractions, analysis of which revealed profound changes in polar lipids caused by HFD intake (Table 3 and Table 4). While AGs and FFAs serve mainly as energy sources [77], alterations of polar lipids, which are the main components of cell membranes and membrane domains, can lead to changes in membrane fluidity, permeability and functions facilitated by the lipid bilayer [83,99]. In mammalian membranes, PUFAs are usually present in the *sn*-2 acyl chain position of PLs, e.g., phosphatidylcholine (PC) and phosphatidylethanolamine (PE) [100]. One consequence of PUFA incorporation into the side chains of membrane lipids is the enhanced fluidity, and this effect increases with the number of double bonds [100,101]. Nonetheless, this comes at the cost of PUFA susceptibility to autooxidation, resulting in damage to DNA or proteins by generation of reactive carbonyl species or induction of inflammation and apoptosis [101]. Our model did not display signs of increased oxidative stress; however, PUFA incorporation into polar lipids suggests a higher risk of membrane damage, which may not be apparent because of the short duration of the study. Moreover, these changes were found not only in phospholipids but also in sphingolipids (Table 4 and Figure 2), which are responsible for cell–cell interactions (GSPL), apoptotic signalling and lipotoxicity (Cer, SM) [83]. PUFA incorporation reduces the thickness and disrupts the geometry of lipid membranes [102] and can disrupt the raft composition, causing displacement of membrane-bound proteins [103]. Since phospholipids are the main cell membrane components, PUFA contains more double bonds than MUFA, and PUFA are more numerous in phospholipids than MUFA (see Table 3), even when total MUFA decrease in heart phospholipids, the simultaneous increase of PUFA content (Table 3) may lead to increased membrane fluidity. Thus, one could speculate that a detrimental effect of increased PUFA content in membrane lipids may be a result of excessive cell membrane fluidity in heart after HFD. Yamamoto et al. [51] reported that an HFD rich in SFAs, but not MUFAs, causes a decrease in the expression of stearoyl-CoA desaturase 1 (SCD1), an enzyme that catalyses the rate-limiting step in MUFA synthesis. It is possible that the heart muscle compensates for the loss of saturation caused by the reduction of SCD1 expression and preferential MUFA β-oxidation by funnelling PUFAs into membrane lipids to alleviate this effect.

Among cardiac lipids, mitochondrial species are particularly worth considering. Heart tissue is rich in mitochondria; therefore, the mitochondrial lipidome accounts for a large portion of total heart lipids, with cardiolipin, a diphosphatidylglycerol PL species unique to mitochondrial membranes, present at approximately 15 nmol/mg protein in mice [104]. Cardiolipin is crucial for the proper function of many proteins in the mitochondrial membrane and is involved in electron flux during ATP production, and within it, the abundance of symmetrical species containing four LA side chains seems to be pivotal to maintaining proper membrane characteristics [105]. In our study, the content of LA in the heart PL fraction was significantly higher in HFD-fed mice (Table 3). However, there are suggestions that cardiolipin LA is readily replaced by longer chain PUFAs, especially DHA [106], which can affect mitochondrial functions [107]. We observed enrichment of DHA in the heart PL fraction (Table 3), but this result was not statistically significant. On the other hand, DPA can replace DHA in some membranes, where its additional bond may influence some membrane-bound protein functions [108]. Under HFD treatment, Sullivan et al. [65] reported the remodelling of some LA-containing cardiolipin species, as well as some mitochondrial phosphatidylcholines and phosphatidylethanolamines, with no significant impairment of mitochondrial supercomplex formation or respiratory enzymatic activity. Thus, the increase in PUFA incorporation in HFD-fed mice may explain the lack of severe cardiac dysfunction observed by us (Table 1).

## 5. Conclusions

In conclusion, the main value of our study was the determination of FA profiles in various lipid groups after HFD treatment of mice, which revealed severe changes in lipids of cell membranes. Considering the preferential β-oxidation of MUFAs in the heart described in the literature, one can expect that it leads to PUFA accumulation after HFD intake and excessive incorporation of PUFAs into membrane lipids. Although HFD caused mild heart dysfunction in our experimental conditions, excessive PUFA incorporation into the cell membrane might change the membrane properties and increase the risk of more serious damage during the progression of obesity. While our data clearly indicate a substantial impact of a high-fat diet on cardiac lipid composition its functional effects still should be treated as preliminary and we feel that work needs to be validated in different, preferably larger, animal cohorts.

## Figures and Tables

**Table 1:** Echocardiographic measurements in C57BL/6 mice fed a standard (SD) or high-fat diet (HFD).

Parameter	SD	HFD	*p*-value
Left ventricular end-systolic volume (LVESV) (µL)	8.80 ± 2.00	10.8 ± 2.40	0.144
Left ventricular end-diastolic volume (LVEDV) (µL)	29.8 ± 4.60	31.1 ± 3.85	0.613
Stroke Volume (SV) (µL)	21.0 ± 2.70	20.3 ± 2.50	0.626
Cardiac Output (CO) (mL × min^-1^)	6.15 ± 0.99	6.10 ± 0.67	0.922
Left Ventricular Ejection Fraction (LVEF) (%)	70.8 ± 4.32	65.4 ± 5.50	0.090
Aortic Valve Area (AVA) (mm^2^)	1.62 ± 0.45	1.34 ± 0.44	0.049
Vmax (m/s)	0.76 ± 0.27	0.98 ± 0.30	0.056

**Table 2:** Composition of selected fatty acids (FAs) in chow, serum and tissues.

	Chow (mg/g)		Serum (mg/L)		Heart (%)		Skeletal muscle (%)	
	10%	60%	SD	HFD	SD	HFD	SD	HFD
16:0	9.73 ± 0.46	74.3 ± 4.89 ^**^	719 ± 210	1106 ± 244 ^*^	21.01 ± 2.82	17.56 ± 2.77 ^*^	23.17 ± 1.78	20.79 ± 1.15
18:0	4.59 ± 0.10	39.1 ± 19.2 ^**^	228 ± 26.3	548 ± 90.4 ^#^	23.88 ± 11.67	21.49 ± 6.64	6.07 ± 2.44	5.10 ± 0.68 ^**^
Other SFAs	3.55 ± 0.10	22.8 ± 1.22 ^**^	101 ± 51.3	201 ± 70.5 ^*^	3.46 ± 0.98	3.01 ± 1.12	5.54 ± 0.35	5.04 ± 0.39
**SFAs**	**17.9 ± 0.64**	**136 ± 8.00** ^**^	**1049 ± 286**	**1855 ± 397** ^**^	**48.4 ± 10.1**	**42.1 ± 6.97**	**34.8 ± 3.50**	**31.0 ± 1.63**
16:1	0.75 ± 0.01	5.93 ± 0.33 ^**^	222 ± 64.9	135 ± 23.7 ^*^	4.07 ± 2.21	1.74 ± 0.87 ^**^	10.8 ± 1.01	9.39 ± 1.02
18:1	9.63 ± 0.14	79.7 ± 3.65 ^#^	920 ± 313	1127 ± 256	30.3 ± 9.60	21.9 ± 7.69 ^*^	45.0 ± 3.03	45.6 ± 3.20
Other MUFAs	0.29 ± 0.01	2.28 ± 0.19 ^**^	21.9 ± 12.0	27.5 ± 8.83	1.13 ± 0.48	0.77 ± 0.32	1.70 ± 0.28	1.03 ± 0.17 ^**^
**MUFAs**	**10.7 ± 0.12**	**87.9 ± 4.15 ^#^**	**1164 ± 389**	**1289 ± 274**	**35.5 ± 12.1**	24.4 ± 8.83 ^*^	**57.5 ± 4.20**	**56.0 ± 2.65**
18:2 *n*-6 (LA)	2.04 ± 0.04	18.6 ± 0.70 ^#^	356 ± 29.1	915 ± 114 ^#^	6.32 ± 1.94	12.2 ± 3.11 ^#^	4.29 ± 0.34	8.78 ± 0.14 ^#^
20:4 *n*-6 (ARA)	0.037 ± 0.004	0.36 ± 0.01 ^#^	152 ± 37.4	330 ± 59.2 ^#^	4.14 ± 2.37	8.62 ± 3.24 ^**^	1.30 ± 0.65	1.46 ± 0.47
22:4 *n*-6 (AdA)	0.008 ± 0.002	0.11 ± 0.02 ^*^	1.48 ± 0.35	2.81 ± 0.60 ^#^	0.18 ± 0.10	0.57 ± 0.27 ^#^	0.12 ± 0.06	0.14 ± 0.03
Other *n*-6 PUFAs	0.073 ± 0.008	0.70 ± 0.08 ^**^	42.0 ± 8.90	47.3 ± 14.2	1.75 ± 0.98	2.19 ± 0.82	0.68 ± 0.22	0.66 ± 0.09
***n*-6 PUFA**	**2.16 ± 0.03**	**19.7 ± 0.64 ^#^**	**552 ± 63.9**	**1295 ± 105 ^#^**	**12.4 ± 4.89**	**23.5 ± 7.17 ^#^**	**6.39 ± 1.02**	**11.0 ± 0.59 ^#^**
18:3 *n*-3 (ALA)	0.005 ± 0.002	0.049 ± 0.002 ^#^	5.74 ± 2.18	9.97 ± 2.55 ^*^	0.02 ± 0.01	0.02 ± 0.01	0.02 ± 0.01	0.03 ± 0.01
20:5 *n*-3 (EPA)	0.008 ± 0.002	0.074 ± 0.004 ^#^	42.9 ± 11.7	20.1 ± 2.77 ^**^	0.54 ± 0.27	0.16 ± 0.05 ^#^	0.34 ± 0.10	0.08 ± 0.01 ^**^
22:5 *n*-3 (DPA)	0.016 ± 0.003	0.15 ± 0.007 ^#^	2.81 ± 0.53	7.87 ± 2.28 ^**^	0.34 ± 0.23	1.87 ± 0.87 ^#^	0.16 ± 0.08	0.36 ± 0.12 ^*^
22:6 *n*-3 (DHA)	0.006 ± 0.003	0.06 ± 0.02	30.9 ± 8.24	72.1 ± 8.81	2.83 ± 1.93	7.91 ± 3.34 ^#^	0.51 ± 0.29	1.10 ± 0.40 ^*^
Other *n*-3 PUFAs	0.002 ± 0.001	0.01 ± 0.01	2.87 ± 0.56	3.44 ± 0.89	0.34 ± 0.23	1.87 ± 0.87 ^#^	0.18 ± 0.09	0.38 ± 0.12 ^*^
***n*-3 PUFA**	**0.04 ± 0.01**	**0.35 ± 0.007 ^#^**	**85.2 ± 21.7**	114 ± 9.01 ^*^	**3.77 ± 2.36**	**9.99 ± 4.24 ^#^**	**1.05 ± 0.47**	**1.59 ± 0.51**
**PUFA**	**2.20 ± 0.03**	**20.1 ± 0.64 ^#^**	**637 ± 82.3**	**1408 ± 112 ^#^**	**16.16 ± 7.14**	**33.53 ± 11.26 ^#^**	**7.44 ± 1.45**	**12.6 ± 1.09 ^#^**

*p*-value from *t*-tests: * *p* < 0.05, ** *p* < 0.01, # < 0.001; AdA–adrenic acid (22:4 *n*-6); ALA–α-linolenic acid (18:3 *n*-3); ARA–arachidonic acid (20:4 *n*-6); DGLA–dihomo-γ-linolenic acid (20:3 *n*-6); DHA–docosahexaenoic acid (22:6 *n*-3); DPA–docosapentaenoic acid (22:5 *n*-3); EPA–eicosapentaenoic acid (20:5 *n*-3); ETA–eicosatetraenoic acid (20:4 *n*-3); LA–linoleic acid (18:2 *n*-6); MUFA–monounsaturated fatty acids, PUFA–polyunsaturated fatty acids; SFA–saturated fatty acids. Bold represents main groups of fatty acids. SD – mice fed standard diet; HFD – mice fed high fat diet.

**Table 3:** FA content (%) in heart fractions obtained with the Kaluzny et al. [62] method.

	Free fatty acids		Phospholipids		Acylglycerols	
	SD	HFD	SD	HFD	SD	HFD
16:0	45.2 ± 4.24	41.4 ± 8.53	21.0 ± 5.57	19.6 ± 4.32	23.6 ± 1.66	22.5 ± 0.64
18:0	28.4 ± 2.24	23.4 ± 3.01 ^**^	27.4 ± 3.32	27.3 ± 3.95	5.30 ± 0.74	9.57 ± 1.25 ^#^
Other SFAs	5.68 ± 1.08	6.19 ± 1.33	2.64 ± 0.83	1.92 ± 0.32	5.55 ± 0.71	6.57 ± 0.86
**SFAs**	**79.3 ± 3.40**	**71.1 ± 11.4**	**51.1 ± 9.40**	**48.8 ± 8.44**	**34.4 ± 1.92**	38.7 ± 1.74 ^**^
16:1	3.22 ± 1.42	3.00 ± 0.70	1.61 ± 0.73	0.65 ± 0.13 ^*^	7.36 ± 1.64	3.84 ± 0.98 ^**^
18:1	13.1 ± 2.80	16.6 ± 6.23	19.8 ± 4.75	13.0 ± 2.06 ^*^	45.7 ± 1.07	38.5 ± 3.90 ^**^
Other MUFAs	0.49 ± 0.19	0.79 ± 0.42	2.29 ± 0.87	1.10 ± 0.12 ^*^	1.80 ± 0.17	1.61 ± 0.29
**MUFAs**	**16.8 ± 2.53**	**20.4 ± 5.94**	**22.2 ± 5.58**	14.2 ± 2.17 ^*^	**54.9 ± 2.20**	43.9 ± 4.89 ^**^
18:2 *n*-6 (LA)	2.48 ± 0.93	5.24 ± 3.00	4.47 ± 0.73	6.24 ± 1.40 ^*^	9.61 ± 3.08	14.3 ± 3.39
20:4 *n*-6 (ARA)	0.53 ± 0.55	1.36 ± 1.08	9.75 ± 5.82	12.7 ± 3.62	0.28 ± 0.24	1.05 ± 0.65 ^*^
22:4 *n*-6 (AdA)	0.11 ± 0.08	0.23 ± 0.19	0.40 ± 0.25	0.71 ± 0.21	0.08 ± 0.07	0.18 ± 0.12
Other *n*-6 PUFAs	0.26 ± 0.16	0.52 ± 0.34	3.52 ± 2.22	2.29 ± 0.61	0.37 ± 0.22	0.81 ± 0.34 ^*^
18:3 *n*-3 (ALA)	0.03 ± 0.02	0.05 ± 0.02	0.03 ± 0.02	0.02 ± 0.01	0.02 ± 0.01	0.03 ± 0.01
20:5 *n*-3 (EPA)	0.18 ± 0.10	0.14 ± 0.07	1.09 ± 0.47	0.19 ± 0.04 ^**^	0.10 ± 0.07	0.10 ± 0.02
22:5 *n*-3 (DPA)	0.12 ± 0.09	0.44 ± 0.43	0.94 ± 0.68	2.84 ± 1.13 ^*^	0.11 ± 0.08	0.39 ± 0.24 ^*^
22:6 *n*-3 (DHA)	0.17 ± 0.12	0.57 ± 0.51	6.51 ± 4.89	12.0 ± 4.20	0.11 ± 0.07	0.60 ± 0.36 ^*^
Other *n*-3 PUFAs	0.001 ± 0.000	0.001 ± 0.000	0.08 ± 0.01	0.06 ± 0.02	0.009 ± 0.003	0.02 ± 0.01
**PUFAs**	**3.88 ± 1.61**	**8.56 ± 5.54**	**26.8 ± 14.9**	**37.1 ± 10.5**	**10.7 ± 3.56**	17.5 ± 3.37 ^*^

*p*-value from *t*-tests: * *p* < 0.05, ** *p* < 0.01, # *p* < 0.001; AdA–adrenic acid (22:4 *n*-6); ALA–α-linolenic acid (18:3 *n*-3); ARA–arachidonic acid (20:4 *n*-6); DHA–docosahexaenoic acid (22:6 *n*-3); DPA–docosapentaenoic acid (22:5 *n*-3); EPA–eicosapentaenoic acid (20:5 *n*-3); LA–linoleic acid (18:2 *n*-6); MUFA–monounsaturated fatty acids; PUFA–polyunsaturated fatty acids; SFA–saturated fatty acids. Bold represents main groups of fatty acids. SD – mice fed standard diet; HFD – mice fed high fat diet.

**Table 4:** Fatty acid content (%) in heart fractions obtained with the Bodennec et al. [63] method.

	Neutral lipids		Ceramides		Glycosphingolipids		Sphingomyelins	
	SD	HFD	SD	HFD	SD	HFD	SD	HFD
16:0	26.2 ± 2.02	24.1 ± 2.08	29.6 ± 4.12	24.3 ± 0.54	43.0 ± 4.14	33.7 ± 0.79 ^*^	24.1 ± 2.13	22.3 ± 1.71
18:0	8.51 ± 1.38	8.59 ± 0.43	16.1 ± 2.74	11.9 ± 1.00 ^*^	34.5 ± 2.80	31.2 ± 1.85	24.6 ± 1.34	25.6 ± 1.29
Other SFAs	6.38 ± 0.62	5.56 ± 0.42	12.9 ± 2.43	8.61 ± 1.81	6.02 ± 2.45	4.64 ± 0.25	2.11 ± 0.12	1.64 ± 0.16 ^**^
**SFAs**	**41.1 ± 1.26**	**38.3 ± 2.33**	**58.6 ± 8.77**	44.8 ± 2.91 ^*^	**83.5 ± 4.68**	69.6 ± 1.43 ^**^	**50.8 ± 3.13**	**49.6 ± 2.97**
16:1	8.28 ± 0.99	3.99 ± 0.58 ^**^	4.98 ± 0.58	4.19 ± 0.69	1.55 ± 0.38	1.53 ± 0.74	1.34 ± 0.09	0.54 ± 0.05 ^#^
18:1	44.0 ± 1.14	42.7 ± 2.02	29.8 ± 7.31	35.5 ± 2.93	9.02 ± 1.70	10.6 ± 0.95	22.6 ± 1.85	14.4 ± 0.52 ^#^
Other MUFAs	2.79 ± 0.63	1.59 ± 0.25	2.52 ± 0.52	1.53 ± 0.23 ^*^	0.31 ± 0.11	0.17 ± 0.01	0.47 ± 0.08	0.28 ± 0.03 ^**^
**MUFAs**	**55.1 ± 1.25**	48.2 ± 2.00 ^*^	**37.3 ± 7.89**	**41.2 ± 2.03**	**10.9 ± 1.32**	**12.3 ± 1.63**	**24.4 ± 1.90**	**15.2 ± 0.57 ^#^**
18:2 *n*-6 (LA)	3.43 ± 1.26	9.70 ± 0.10 ^**^	2.71 ± 0.82	8.86 ± 1.27 ^#^	1.74 ± 0.68	4.20 ± 0.30 ^**^	6.00 ± 0.96	8.15 ± 1.55 ^*^
20:4 *n*-6 (ARA)	0.30 ± 0.27	1.20 ± 0.14 ^*^	0.75 ± 0.35	1.99 ± 0.29 ^**^	2.21 ± 1.34	6.63 ± 0.21 ^**^	9.07 ± 1.66	12.4 ± 1.09 ^*^
22:4 *n*-6 (AdA)	0.07 ± 0.05	0.47 ± 0.06 ^**^	0.05 ± 0.03	0.37 ± 0.02 ^#^	0.07 ± 0.04	0.36 ± 0.01 ^#^	0.33 ± 0.07	0.70 ± 0.07 ^#^
Other *n*-6 PUFAs	0.19 ± 0.12	0.90 ± 0.17 ^**^	0.23 ± 0.09	0.59 ± 0.11 ^**^	0.50 ± 0.40	0.90 ± 0.10	2.98 ± 0.71	2.34 ± 0.14
18:3 *n*-3 (ALA)	0.02 ± 0.01	0.02 ± 0.01	0.06 ± 0.02	0.04 ± 0.01	0.04 ± 0.02	0.02 ± 0.01	0.01 ± 0.01	0.02 ± 0.01
20:5 *n*-3 (EPA)	0.22 ± 0.15	0.16 ± 0.04	0.4 ± 0.23	0.18 ± 0.06	0.43 ± 0.17	0.09 ± 0.02 ^*^	1.39 ± 0.24	0.18 ± 0.02 ^#^
22:5 *n*-3 (DPA)	0.12 ± 0.10	1.01 ± 0.14 ^**^	0.08 ± 0.04	0.88 ± 0.07 ^#^	0.15 ± 0.12	1.35 ± 0.16 ^#^	0.72 ± 0.20	2.57 ± 0.39
22:6 *n*-3 (DHA)	0.12 ± 0.08	0.84 ± 0.12 ^**^	0.32 ± 0.13	1.39 ± 0.07 ^#^	1.07 ± 0.86	4.81 ± 0.23 ^#^	4.75 ± 1.43	9.08 ± 0.64 ^#^
Other *n*-3 PUFAs	0.013 ± 0.005	0.02 ± 0.01	0.010 ± 0.007	0.03 ± 0.01 ^*^	0.02 ± 0.01	0.04 ± 0.01	0.10 ± 0.02	0.09 ± 0.03 ^#^
**PUFA**	**4.47 ± 2.01**	14.3 ± 0.70 ^**^	**4.61 ± 1.61**	**14.3 ± 1.04 ^#^**	**6.24 ± 3.57**	18.4 ± 0.37 ^**^	**25.4 ± 5.05**	35.5 ± 3.43 ^*^

*p*-value from *t*-tests: * *p* < 0.05, ** *p* < 0.01, # *p* < 0.001; AdA–adrenic acid (22:4 *n*-6); ALA–α-linolenic acid (18:3 *n*-3); ARA–arachidonic acid (20:4 *n*-6); DHA–docosahexaenoic acid (22:6 *n*-3); DPA–docosapentaenoic acid (22:5 *n*-3); EPA–eicosapentaenoic acid (20:5 *n*-3); LA–linoleic acid (18:2 *n*-6); MUFA–monounsaturated fatty acids; PUFA–polyunsaturated fatty acids; SFA–saturated fatty acids. Bold represents main groups of fatty acids. SD – mice fed standard diet; HFD – mice fed high fat diet
